# Construction and immunological evaluation of recombinant adenovirus vaccines of new novel NADC34-PRRSV strains in pigs

**DOI:** 10.3389/fvets.2024.1503526

**Published:** 2024-12-17

**Authors:** Chang-zhan Xie, Ping Zhang, Zheng Wang, Yi-mo Tao, Zhuo-dong Cui, Fu-long Nan, Fu-chao Zhang, Yun-xin Ren, He Zhang, Hui-jun Lu

**Affiliations:** ^1^Institute of Urban Agriculture, Chengdu National Agricultural Science & Technology Center, Chinese Academy of Agricultural Sciences, Chengdu, China; ^2^Key Laboratory of Special Animal Epidemic Disease, Institute of Special Animal and Plant Sciences, Chinese Academy of Agricultural Sciences, Changchun, China; ^3^Changchun Veterinary Research Institute, State Key Laboratory of Pathogen and Biosecurity, Key Laboratory of Jilin Province for Zoonosis Prevention and Control, Chinese Academy of Agricultural Sciences, Changchun, China; ^4^College of Animal Science and Veterinary Medicine, Southwest Minzu University, Chengdu, China

**Keywords:** PRRSV, epidemiological investigation, vaccine, construction, immunogenicity

## Abstract

**Introduction:**

Porcine reproductive and respiratory syndrome virus (PRRSV) causes reproductive and respiratory diseases in sow herds and piglets. The emergence of ORF5 RFLP 1–7-4-like (NADC34-like) PRRSV strain in China has brought a new round of challenges to PRRSV prevention.

**Methods:**

In addition, recombinant adenovirus vaccine candidates against the newly emerged NADC34-like strain were constructed in the study; the immunogenicity of the vaccine was investigated in piglets. After inoculation with PRRSV recombinant adenovirus, specific antibodies, neutralizing antibodies, and levels of IFN-*γ* and IL-4 cytokines were detected in serum.

**Results:**

Thirty-five days after immunization, the levels of IFN-γ and IL-4 cytokines in the pac-Ad5-34-GP3, pac-Ad5-34-GP5, and pac-Ad5-34-GP35 experimental groups were significantly higher (*p* < 0.05) than those of the PBS and the adenovirus group. All vaccines can cause corresponding Th1 and Th2 immune responses based on animal experimental results. After the challenge, no obvious clinical symptoms were observed in the immune groups compared with the control group, vaccinated animals could reduce the occurrence of viremia, and the occurrence of viremia was alleviated, with no obvious pathological changes in the lungs, indicating that recombinant adenovirus vaccine could provide a good protective immunity and produce a good humoral and cellular immune response at the same time.

**Discussion:**

It shows that the recombinant adenovirus vaccine group has better protection against the virus. Provide vaccine reserve and theoretical support for the emergence of new PRRSV subtypes in China.

## 1 Introduction

Porcine reproductive and respiratory syndrome virus (PRRSV) causes huge losses to the pig industry every year. The main reason is that PRRSV has highly evolved genetic characteristics (1, 2). PRRSV is a member of Arteriviridae Nidovirales (a species of Arteriviridae Nidovirales) and has two main genotypes: PRRSV type 1 (European type) and PRRSV type 2 (North American type) (2, 3). According to the phylogenetic analysis of ORF5, PRRSV-2 is further divided into 9 lineages (3). CH-1a of PRRSV-2 strain of lineage 8 in China was first classified in 1996 (4). In 2006, the highly pathogenic PRRSV (HP-PRRSV) strain (lineage 8) had consecutive deletions of 30 amino acids in its NSP2, and in the following years, it became the main PRRSV strain in China (5). Since 2013, a new type of NADC30-like PRRSV-2 strain (lineage 1) has been isolated in several provinces in China. Compared with VR2332, its Nsp2 has a discontinuous deletion of 131 amino acids (6, 7). According to the analysis of lineage and system dynamics, the representative isolate QYYZ of lineage 3 PRRSV strain was first reported in Taiwan and appeared in Hong Kong, China, in 2004 (8–10). In 2011, a QYYZ-like strain named GM2 was isolated from China and proved to be a recombinant strain between QYYZ and VR2332 MLV (9, 11, 12). In 2017, the NADC34-like strain was reported for the first time in Shenyang, China, and followed by reports in Heilongjiang and Fujian.

In this study, we successfully rescued NADC34 recombinant adenovirus vaccines pac-Ad5-34-GP3, pac-Ad5-34-GP5, and pac-Ad5-34-GP35. After the verification is correct, the piglet immunogenicity and challenge protection experiments are carried out, and the immunogenicity of the recombinant adenovirus candidate vaccine is evaluated by detecting the cytokines, specific antibodies, and neutralizing antibodies in the serum of the immunized piglets.

## 2 Materials and methods

### 2.1 Viruses and cells

HEK293 and MARC-145 cells were grown in DMEM containing 10% fetal bovine serum (FBS) and 1% streptomycin. The NADC34-like PRRSV strain was separated in this laboratory. The virus titer was 1 × 10^3.5^ TCID_50_/ml.

### 2.2 Amplification of GP3 and GP5 of NADC34-like PRRSV

In order to amplify the GP3 and GP5 genes of MADC34-like, PCR primers were designed (Table 1). Gene synthesis after optimization design (Sangon Biotech (Shanghai)). Three recombinant adenoviruses (pac-Ad5-34-GP3, pac-Ad5-34-GP5, and pac-Ad5-34-GP35) were constructed, and the sequence encoding the G4S flexible linker was inserted into the adenovirus expression plasmid pac-Ad5-34-GP35 between the GP3 and GP5 genes.

**Table 1:** Primers of PCR amplification.

Primer name	Sequence (5′-3′)	Amplification size
GP3(34)-F	ATGGCTAATAGCTGTACACTCCTCCA	765 bp
GP3(34)-R	CTATCGCCGTACGGCACTGAGGGAT	
GP5(34)-F	ATGTTGGAGAAATGCTTGA	603 bp
GP3(34)-F	CTAAAGACGACCCCATTGTTCCGCTGA	

### 2.3 Western blot

The successfully packaged recombinant adenovirus was used to inoculate a six-well plate containing 70% HEK293 cells. When the cells showed CPE, they were recovered and sonicated, and the supernatant was recovered after centrifugation and separated by 10% SDS-PAGE. The protein is transferred to a polyvinylidene fluoride (PVDF) membrane (Millipore, Billerica, USA) and blocked (5% BSA), the membrane is combined with PRRSV-specific GP5 antibody (diluted 1:100 in PBS containing 0.05% Tween-80, PBST), incubated at 37°C for 2 h, washed the membrane, and then incubated with goat anti-rabbit IgG horseradish peroxide (HRP) secondary antibody at 37°C for 1 h. The results were observed using GE Healthcare Bio-Sciences AB.

### 2.4 Vaccination of pigs with recombinant adenovirus vaccine and challenged by NADC34-like PRRSV

Thirty-five weaned piglets of 4–5 weeks were randomly divided into seven groups, each with 5 pigs. From the first to the sixth group, they were immunized with recombinant adenovirus pac-Ad5-34-GP3, pac-Ad5-34-GP5 and pac-Ad5-34-GP35, as well as the control group HP-PRRSV commercial vaccine, ad-wt (adenovirus wild virus) and PBS negative control, the immunization dose was shown in Table 2; booster immunization on the 21st day; 35 days after immunization, NADC34 strain challenge protection test (Table 2).

**Table 2:** Group and immunogenicity trial in pig by adenovirus vaccines.

Group	First immunization	Second immunization	Immunization dose	Quantity	Virus strain
1	pac-Ad5-34-GP3	pac-Ad5-34-GP3	1.0 × 10^8^TCID_50_/2 mL	5	NADC34
2	pac-Ad5-34-GP5	pac-Ad5-34-GP5	1.0 × 10^8^TCID_50_/2 mL	5	NADC34
3	pac-Ad5-34-GP35	pac-Ad5-34-GP35	1.0 × 10^8^TCID_50_/2 mL	5	NADC34
4	HP-PRRSV (Inactivated vaccine)	HP-PRRSV (Inactivated vaccine)	1.0 × 10^8^TCID_50_/2 mL	5	NADC34
5	ad-wt	ad-wt	1.0 × 10^8^TCID_50_/2 mL	5	NADC34
6	PBS	PBS	2 mL	5	NADC34

### 2.5 Specific antibody detection

The pig serum was tested for specific antibodies. The inactivated standard antigen of GP5 subtype was coated overnight in a 96-well plate (expression and purification in the laboratory). After blocking with 5% skim milk for 2 h, the samples were washed three times with PBST buffer for 5 min each time; added the sample to be tested (5 μL/well) to the ELISA plate, incubated at 37°C for 2 h, and washed three times with PBST buffer for 5 min each time; 1:1000 diluted HRP-labeled goat anti-mouse IgG secondary antibody (100 μL/well) to the ELISA plate was added, incubated at 37°C for 2 h, washed three times with PBST buffer, 5 min each time; TMB chromogenic substrate (100 μL/well) was added and protected from light and room temperature for 10 min; stop solution was added to the ELISA plate to stop the reaction (50 μL/well) and measured the OD value at 450 nm absorbance.

### 2.6 Neutralizing antibody detection

The porcine serum was inactivated in a 56°C water bath for 30 min and centrifuged at 3000 rpm for 10 min; DMEM culture medium was used to dilute the inactivated serum with a 2-fold gradient and added it to a 96-well plate, with three replicate wells for each gradient; 200 TCID_50_ NADC34-like was added to each well virus solution, set up positive and negative controls. After incubation at 37°C for 2 h, 3 × 103 MARC-145 cells were added to each well and were cultured at 37°C and 5% CO_2_ for 5–7 days to observe the cell CPE phenomenon. Spearman–Karber calculation was made.

### 2.7 Cytokines secretion assay

Serum IL-4 and IFN-*γ* was detected according to the manufacturer’s instructions (ELISA Ready-SET-Go!^®^, eBioscience, San Diego, CA, USA).

### 2.8 Measurements of viremia and the tissue virus loads in pigs

On the 35th day after immunization, the vaccine immunization experimental group and the control group were subjected to a PRRSV challenge protection test. The challenge titer was 1.0 × 10^3^ TCID_50_/mL, and each piglet was injected with 2 mL into the neck muscle. Pig rectal temperatures were measured daily during challenge; 14 days after the challenge, the piglet’s heart, liver, spleen, lung, kidney, submandibular lymph node, and inguinal lymph node were ground to extract RNA, and a reverse transcription kit (TaKaRa Biotechnology, Dalian, China) was used according to the manufacturer’s instructions. Reverse transcriptase nested polymerase chain reaction (RT-PCR) was used to amplify the PRRSV ORF7 gene, and a quantitative fluorescence detection method (absolute quantification) was established to detect the PRRSV viral load in blood and tissues. Referring to the sequence of the PRRSV ORF7 gene, primers 5′-ATGGCCAGCCAGTCAATCA-3′ and 5′-TCGCCCTAATTGAATAGGTG-3′ were used to design primers. The reaction was carried out at 95°C for 5 min, followed by 35 cycles of 95°C for 45 s and 53°C for 45 s.

### 2.9 Pathology of lung tissue after challenge

The dissected lung tissues were fixed with 4% formalin solution, and the fixed samples were dehydrated and coated with paraffin and then sectioned and stained (Servicebio®, Wuhan, China).

### 2.10 Statistical analysis

All data were analyzed using GraphPad Prism software (version 6.0) and expressed as mean ± SD. The cytokine data were evaluated by one-way repeated-measures analysis of variance. The difference was considered significant (*p* < 0.05).

## 3 Results

### 3.1 Western blot detection of recombinant proteins GP3 and GP5 proteins expression from NADC34-like PRRSV

**Figure 1:**
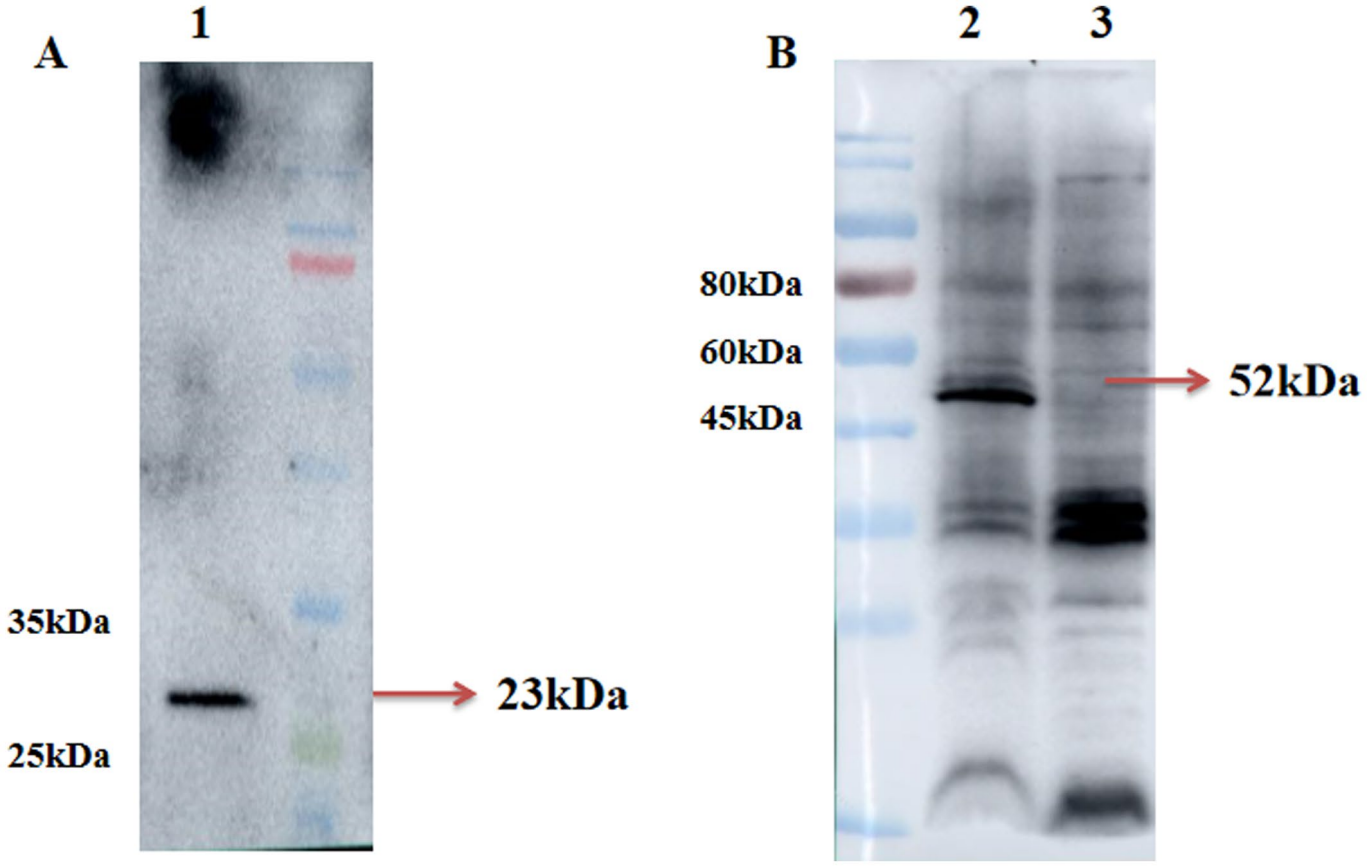
Identification of rAd5-34-GP5 and rAd5-34-GP35 by Western blot. **(A)** Detection of GP5 after infected pac-Ad5-34-GP5; **(B)** Detection of GP5 after infected pac-Ad5-34-GP35. Data are presented as mean ± SE.

Recombinant adenovirus pac-Ad5-34-GP5 and pac-Ad5-34-GP35 can detect GP5 and GP3-GP5 protein expression after infection of HEK293 cells were 23 kDa and 52 kDa, respectively. The control group did not express the target protein (Figure 1).

### 3.2 Detection of specific antibodies in immune pigs

**Figure 2:**
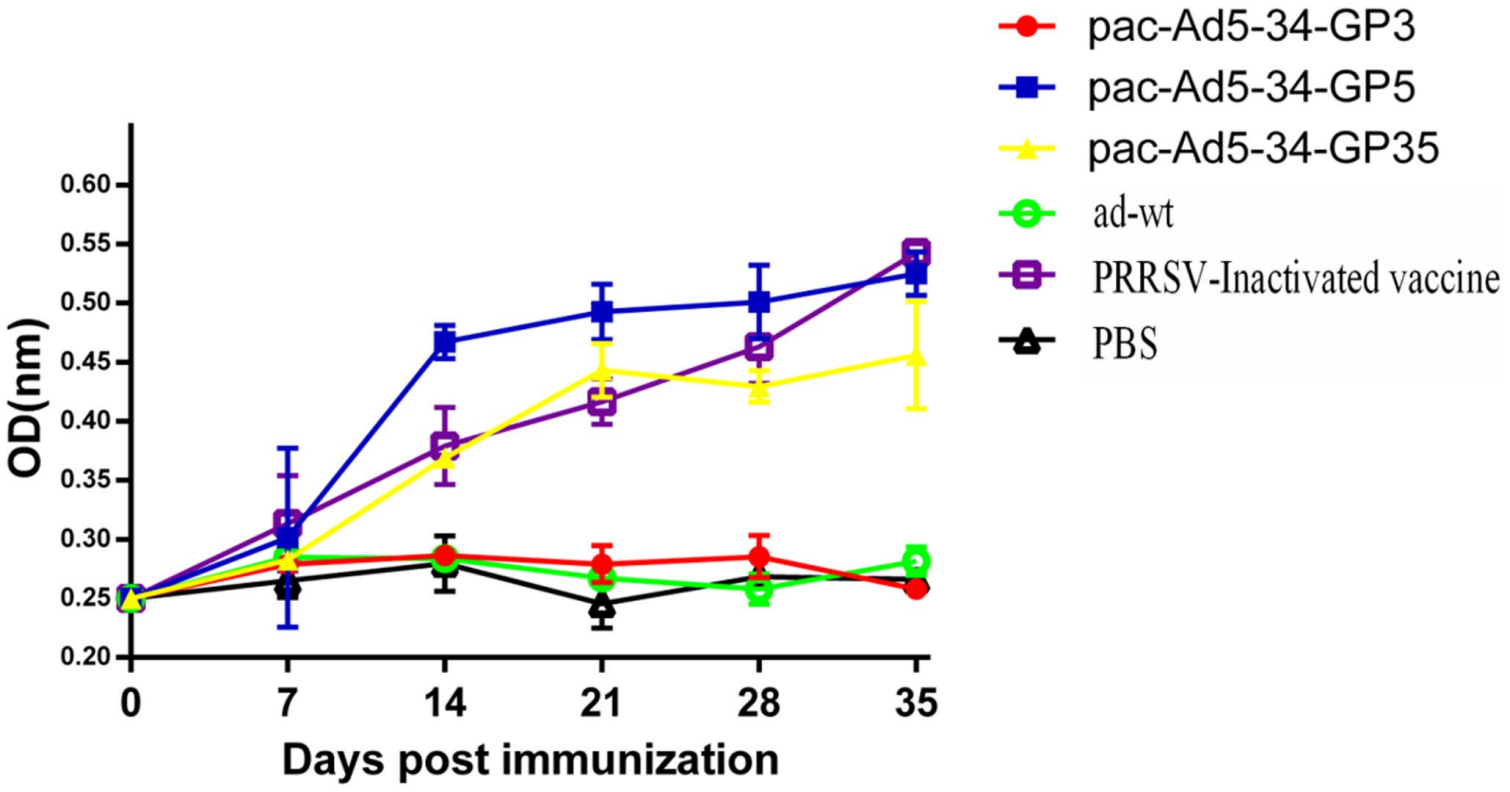
Porcine reproductive and respiratory syndrome virus (PRRSV)-specific GP5 antibody responses in the groups of pigs immunized with the recombinant adenoviruses vaccines. Data are presented as mean ± SE.

After immunization, blood was collected every week to detect PRRSV GP5-specific antibodies. The results showed that the recombinant adenovirus vaccine pac-Ad5-34-GP3 experimental group, adenovirus wild virus control group, and PBS control group did not produce GP5-specific antibodies. Adenovirus vaccines pac-Ad5-34-GP5 and pac-Ad5-34-GP35 experimental group and commercial vaccine control group produced corresponding GP5-specific antibodies, but the difference between the groups was not significant (*p* > 0.05). On the 35th day after immunization, the recombinant adenovirus vaccine pac-Ad5-34-GP5 experimental group had the highest GP5 antibody level than other experimental groups (Figure 2).

### 3.3 Detection of neutralizing antibodies in sera

**Figure 3:**
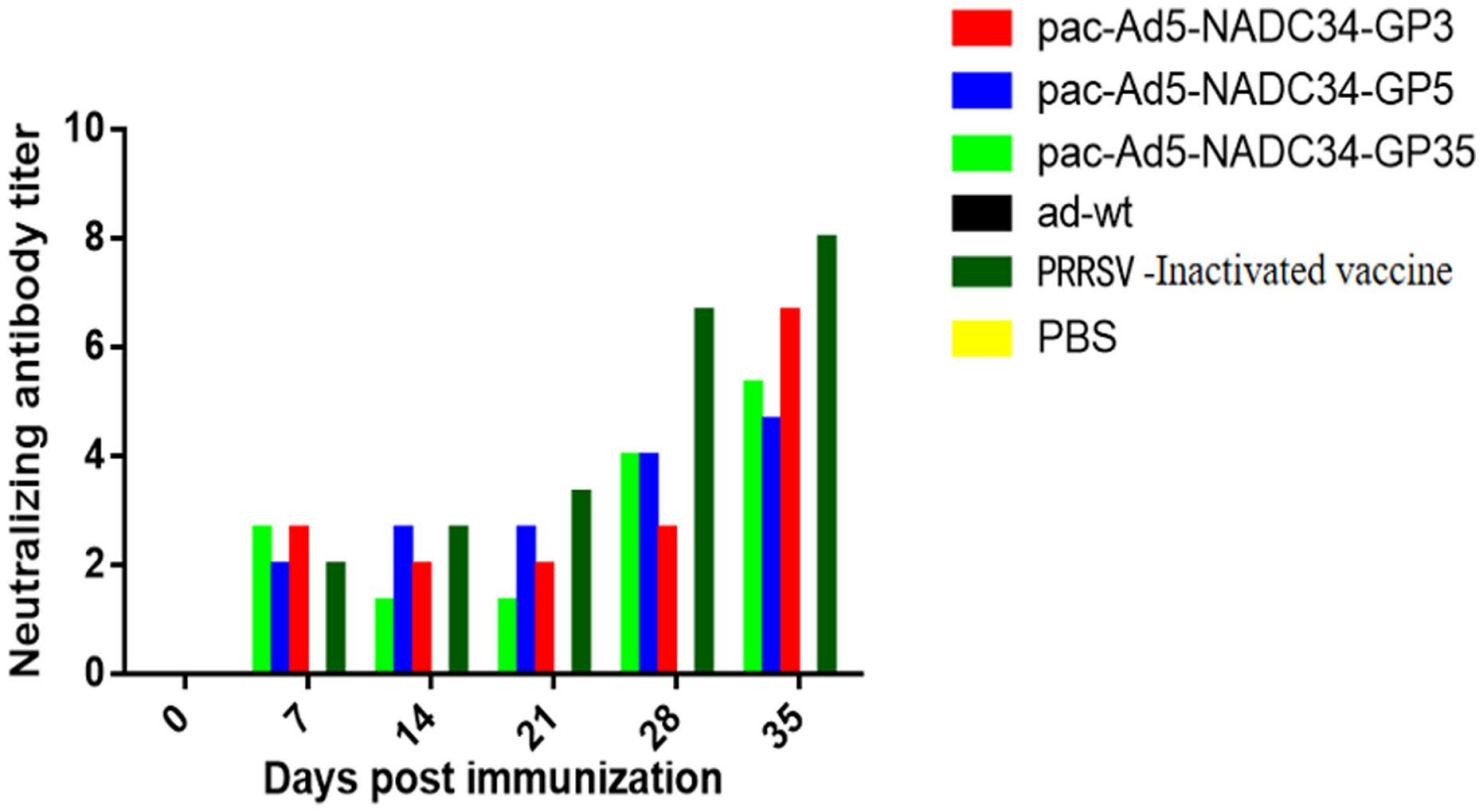
Anti-porcine reproductive and respiratory syndrome virus (PRRSV) neutralizing antibodies in pigs inoculated with the recombinant adenovirus vaccines.

Serums were tested for PRRSV-neutralizing antibodies at different time points after immunization. The results showed that neutralizing antibodies appeared in the recombinant adenovirus vaccine experimental group on the 28th day after immunization, but the adenovirus wild virus control group and the PBS control group never produced neutralizing antibodies. On the 35th day after immunization, the commercial vaccine immunization experimental group has a higher neutralizing titer than other immunization groups, and the difference between the groups is not significant (*p* > 0.05; Figure 3), indicating that the recombinant adenovirus vaccine can produce certain neutralizing antibodies in pigs (Table 3).

**Table 3:** Anti-PRRSV neutralizing antibodies in pigs inoculated with the recombinant adenovirus vaccines.^a^

Immune group	7dpi^b^	14dpi	21dpi	28dpi	35dpi
rAd-34-GP3	-^c^	-	2.00 ± 0	7.80 ± 0.45	10.95 ± 1.50
rAd-34-GP5	-	5.62 ± 1.85	6.50 ± 0.83	9.56 ± 1.89	12.39 ± 0.81
rAd-34-GP35	-	7.80 ± 2.34	8.83 ± 0.61	12.61 ± 1.94	16.34 ± 0.39
HP-PRRSV (Inactivated vaccine)	-	8.56 ± 1.00	9.89 ± 1.66	13.34 ± 2.11	19.28 ± 2.94
PBS	-	-	-	-	-
Wild-type adenovirus	-	-	-	-	-

a Serum samples from vaccinated piglets were individually analyzed to determine the neutralizing antibody titers.

b The number of days after primary vaccination (days post challenge).

c Neutralizing antibodies are negative or the titer is less than 1:2.

### 3.4 Levels of secreted cytokines IL-4 and IFN-*γ* after immunization

The serum of piglets were tested for IFN-*γ* and IL-4 cytokines at 14 and 35 days after immunization.

**Figure 4:**
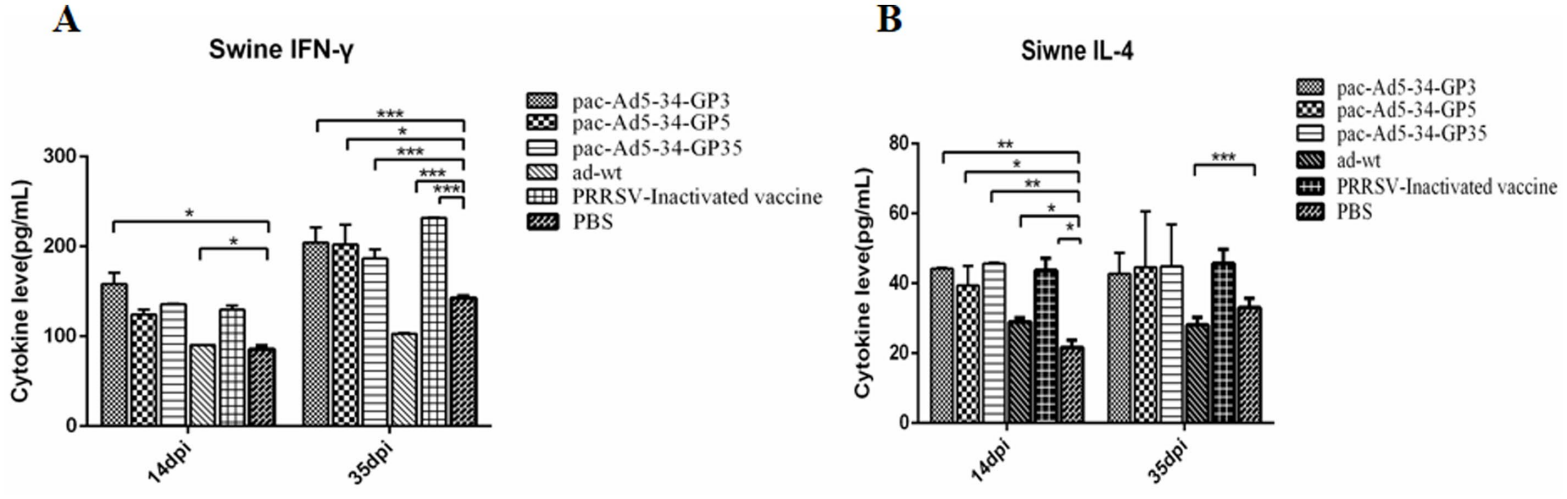
Detection of the level of cytokine secretion in the serum of the immunized pigs. The mean concentrations (pg/ml) of Th1-type cytokine IFN-*γ* **(A)** as well as the Th2 cytokineIL-4 **(B)** in the serum of the immunized pigs. Data are presented as mean ± SE.

The results showed that the expression level of cytokine IFN-γ in the immune group increased at 35dpi compared with 14dpi, which may be the reason for the booster immunity. The expression level of IFN-*γ* cytokine in the vaccine experimental group was higher than that in the control group; 35 days after immunization, the expression levels of IFN-γ cytokine of the pac-Ad5-34-GP3 and pac-Ad5-34-GP5 experimental groups were 1.99, 1.97 times and 1.42, 1.43 times that of the PBS control group and the adenovirus wild virus control group, respectively, the difference was significant (*p* < 0.05); the IFN-γ cytokine expression levels in the pac-Ad5-34-GP35 experimental group were 1.82 times and 1.31 times higher than those in the PBS control group and the adenovirus wild-virus control group, respectively. The differences were significant (*p* < 0.05; Figure 4A).

The IL-4 cytokine expression levels in the pac-Ad5-34-GP3 and pac-Ad5-34-GP5 experimental group were 1.52, 1.59 times and 1.60, 1.92 times higher than those in the PBS control group and the adenovirus wild-virus control group, respectively. The differences were significant (*p* < 0.05). The IL-4 cytokine expression levels in the pac-Ad5-34-GP35 experimental group were 1.30 times and 1.36 times higher than those in the PBS control group and the adenovirus wild-virus control group, respectively. The differences were significant (*p* < 0.05). Compared with the experimental groups of pac-Ad5-34-GP3, pac-Ad5-34-GP3, and pac-Ad5-34-GP35, the difference was not significant (*p* > 0.05; Figure 4B). The increase in IFN-*γ* cytokine content indicates that piglets can effectively secrete Th1 cytokines, the level of cellular immunity is enhanced, and PRRSV infection can be inhibited. The increase in IL-4 cytokine expression level indicates that piglets can effectively secrete Th2 cytokine, which has a certain effect on the enhancement of humoral immunity.

### 3.5 Viremia and tissue-specific viral loads after PRRSV challenge

**Figure 5:**
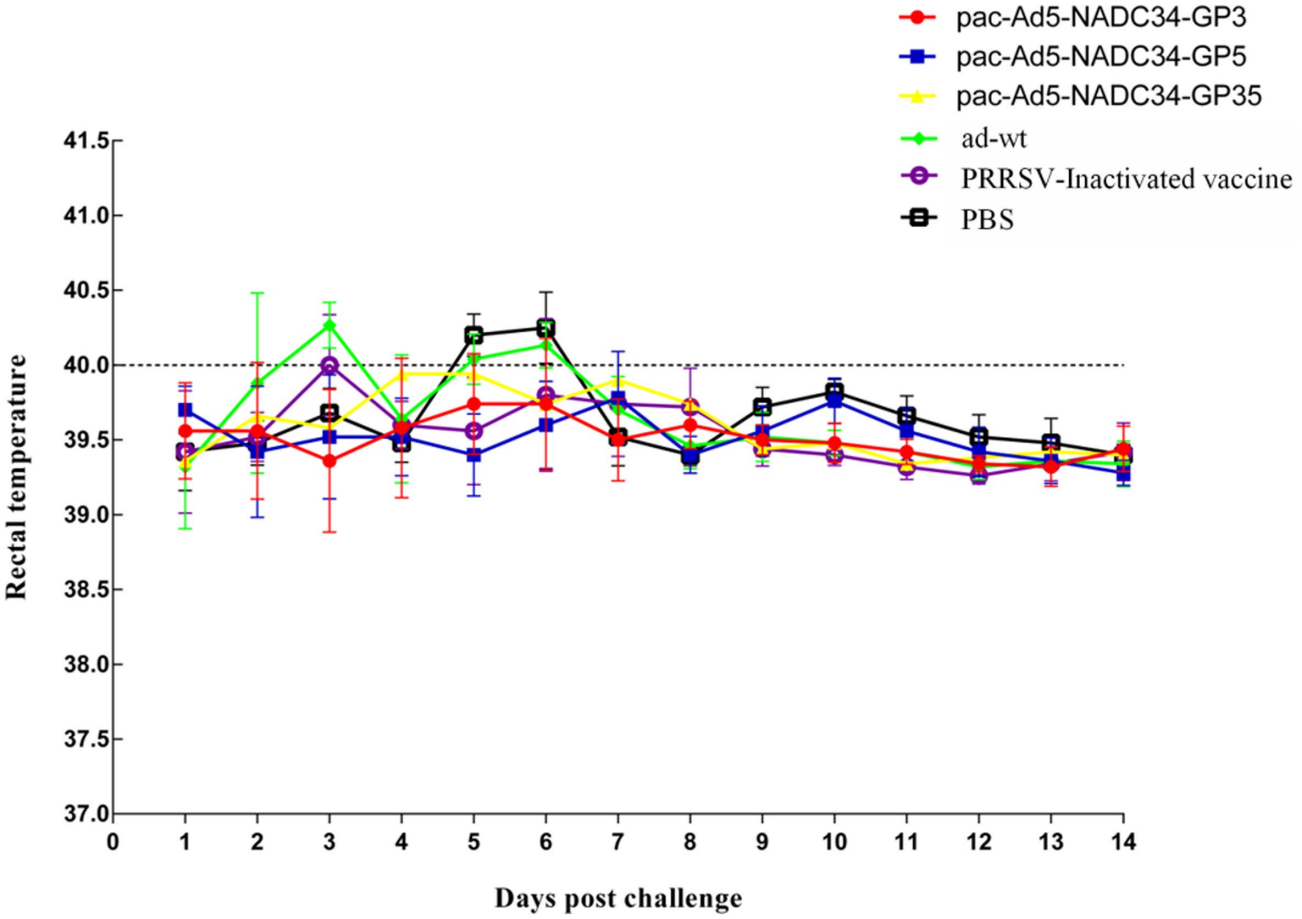
Changes in temperature post challenge of the PRRSV strain (NADC34-like). Data are presented as mean ± SE.

**Figure 6:**
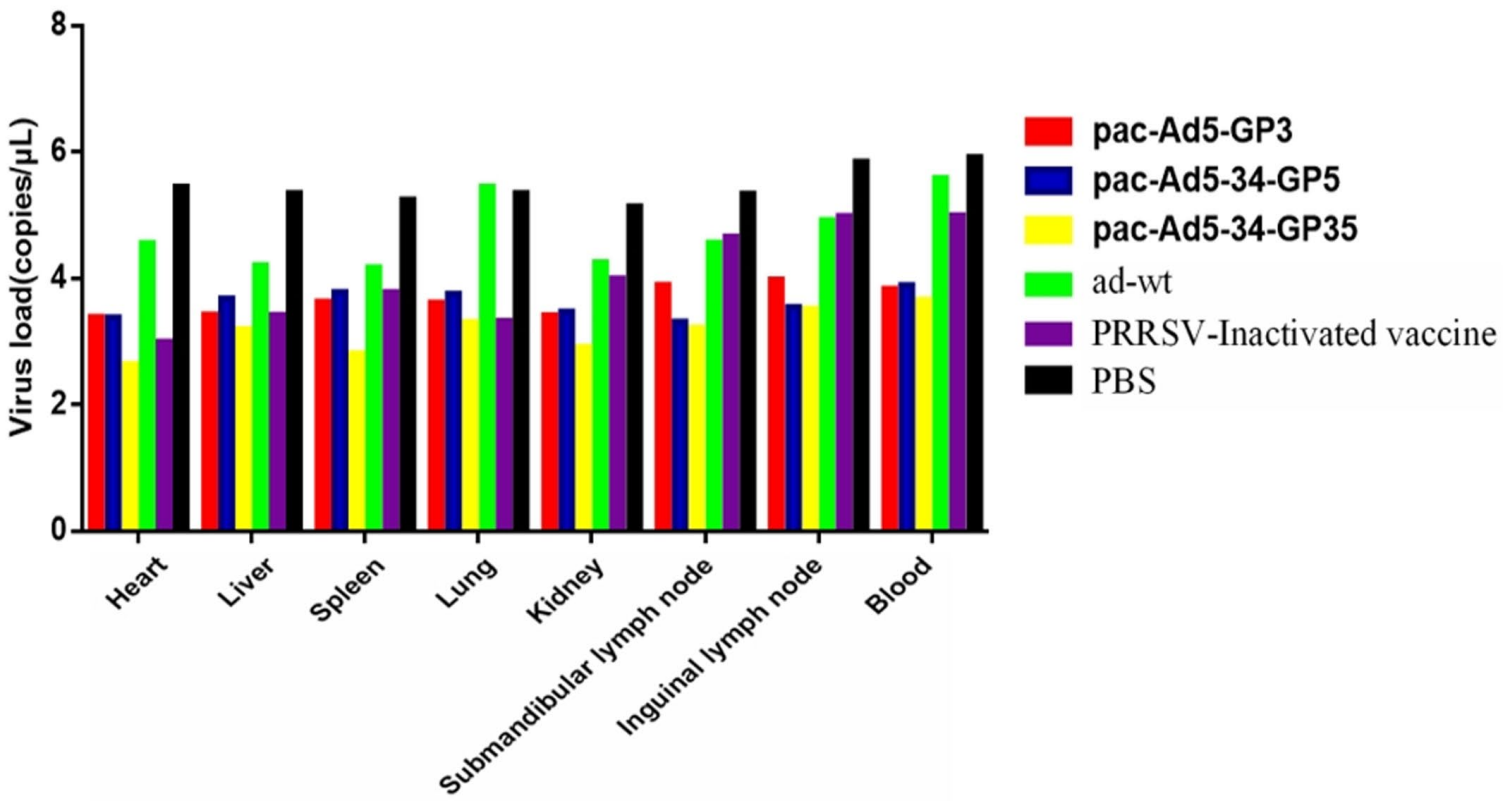
Viral loaded in the tissues of the pigs inoculated with recombinant adenovirus followed by a challenge with the PRRSV strain (NADC34-like). Data are presented as mean ± SE.

The body temperature of the NADC34 strain changes after the challenge; 3 days after the challenge, the body temperature of the adenovirus control group began to rise and reached 40.4°C; the body temperature of the PBS control group started to rise on the 6-7th day, reaching 40.3°C and 40.4°C, and then began to decrease it tends to be normal. The body temperature of the adenovirus control group and the PBS control group after the challenge was slightly higher than the other vaccine-immunized groups, and the other experimental groups did not experience any increase in body temperature (Figure 5). On the 14th day after the challenge, the piglets were subjected to necropsy and the main tissues and organs were analyzed for viral load. The results showed that the experimental group had a lower viral load in the tissues and organs and also lower than the adenovirus control group and the PBS control group in terms of viremia. According to the viremia, vaccine immunity compared with the control group showed that the group decreased by approximately 2.5 titers (Figure 6). It shows that the recombinant adenovirus vaccine group has a better protection effect against the virus.

### 3.6 Macroscopic and microscopic lesions in the lungs

**Figure 7:**
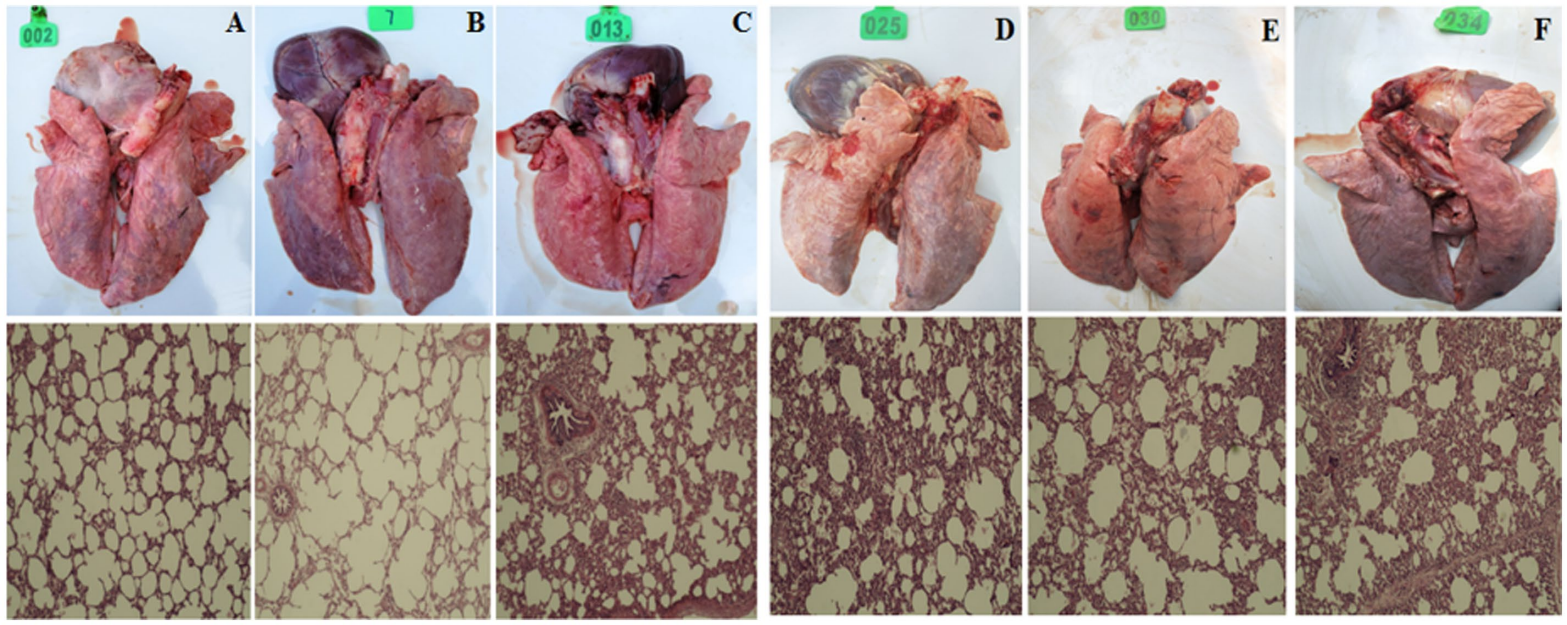
Clinical anatomy and histopathological examination pictures of Lung. **(A)** pac-Ad5-34-GP3; **(B)** pac-Ad5-34-GP5; **(C)** pac-Ad5-34-GP35; **(D)** wild adenovirus group; **(E)** commercial vaccine group; **(F)** PBS control group (200x).

After 14 days of challenge, the piglets were dissected and the lungs were examined for histopathology. Clinical manifestations: pac-Ad5-34-GP3, pac-Ad5-34-GP5, and pac-Ad5-34-GP35 experimental groups did not show clinical symptoms such as loss of appetite and mild cough. The adenovirus wild virus control group and the PBS control group showed general clinical symptoms after challenge, including difficulty breathing and coughing. Histopathological analysis showed that the pac-Ad5-34-GP3, pac-Ad5-34-GP5, and pac-Ad5-34-GP35 experimental groups showed local alveolar wall thickening, accompanied by a small amount of lymphocyte infiltration, and a small amount of lymphocyte infiltration and showed PRRSV lung pathology score level 2. The adenovirus wild virus control group and the PBS control group showed more alveolar wall thickening, accompanied by more lymphocyte infiltration and large alveolar cavity expansion and showed PRRSV lung pathology score level 4 (Figure 7).

## 4 Discussion

In recent years, swine diseases caused by the NADC30-Like PRRSV strain have broken out in China. PRRSV is a single-stranded positive-stranded RNA virus with a genome length of approximately 15 kb, which contains at least 10 open reading frames (ORF), including ORF1a, ORF1b, ORF2a, ORF2b, ORF3, ORF 4, ORF 5, ORF 5a, ORF 6, and ORF 7 (13, 14). In China, the PRRSV-2 genotype is widespread and has had a significant impact on the pig industry in the past few years. According to the global PRRSV classification, the PRRSV genotype 2 strains in China are divided into four gentypes: JXA1-like/CH-1a-like (Lineage 8), VR2332-like (Lineage 5), QYYZ-like (Lineage 3), and NADC30-like (Lineage 1) (15, 16). Recently, PRRSV-ORF5-RFLP 1–7-4 lineage (NADC34-like) isolates appeared in the United States, and both sow herds and piglets showed relatively high mortality (17). The ORF5 RFLP 1–7-4-like (NADC34-like) PRRSV strain was first reported in China (Shenyang) in 2017, and it was first reported in Peru in 2019.

PRRSV is still the most economically important disease problem in the world’s pig industry. Commercial attenuated PRRSV vaccines usually only have a limited effect on limited PRRSV virulent attacks (11, 12) and have the risk of reversion to virulence (18). Therefore, genetic engineering vaccines have been reported, including recombinant baculovirus expressing GP3 or GP5 protein (19), *Mycobacterium bovis* BCG expressing GP5 and M protein (20, 21), rabies virus expressing GP5 protein and recombinant fowl-pox virus of GP5/GP3 and porcine IL-18 (22). In order to improve the efficiency of the vaccine, another method is to co-deliver cytokines to upregulate the immune response of vaccine antigens, including IL-1 (23), IL-2 (24), IL-12 (25), and GM-CSF (26, 27). GM-CSF is a pleiotropic cytokine that has been used as an adjuvant to enhance the immune response of many vaccine antigens. As compared with standard SIV VLP (28), mice immunized with chimeric SIV VLP containing GM-CSF showed a significant increase in CD4 ^+^ and CD8 ^+^ T-cell responses to SIV Env (29).

So far, adenoviral vectors are still one of the most popular gene transfer vectors used in gene-based clinical trials (approximately 1/3), and are mainly used for angiogenesis applications and cancer immunotherapy research (30, 31). Recently, adenovirus vectors have been used as vaccine vectors against infectious diseases, and several adenovirus vector vaccines have been developed into non-human primate studies (32, 33). It was found that the human replication-deficient adenovirus type 5 (Ad5) vaccine expressing SIVgag can trigger an effective antigen-specific antibody and CD8^+^ T-cell response in an experimental model and provide protection in a primate challenge model (34).

The results of pig immunogenicity in this study showed that the pac-Ad5-34-GP5 and pac-Ad5-34-GP35 experimental groups and the commercial vaccine control group produced corresponding GP5-specific antibodies. On the 35th day after immunization, the GP5 antibody level of the pac-Ad5-34-GP5 experimental group was the highest compared with other experimental groups. On the 35th day after immunization, the commercial vaccine-immunized experimental group had a higher neutralizing titer than other immunized groups, indicating that the recombinant adenovirus vaccine can produce certain neutralizing antibodies in pigs, and the neutralizing antibody level of the experimental group was significantly lower than that of other immunized groups. The commercial vaccine group corresponds to the results of Th1 cytokine and Th1 cytokine secretion after immunization, which may be caused by the low expression of recombinant adenovirus vaccine membrane protein. The stimulation index of the experimental group pac-Ad5-34-GP3, pac-Ad5-34-GP5 and pac-Ad5-34-GP35 were 1.15, 1.2, 1.14 times and 1.53, 1.58, 1.51 times that of the adenovirus control group and the PBS control group (significant difference *p* < 0.05); indicating that the experimental group can be better promote the proliferation of lymphocytes. These results indicate that the recombinant adenovirus vaccine expressing GP3 and GP5 proteins alone and the recombinant adenovirus vaccine expressing GP3 and GP5 proteins at the same time can induce cellular immunity and humoral immunity, respectively.

The results of the challenge protection test of recombinant adenovirus vaccine after immunization showed that the body temperature of the American type NADC34 strain changed after the challenge. 3 days after the challenge, the body temperature of the adenovirus control group began to rise and reached 40.4°C; the PBS control group was on the sixth and seventh celestial body temperature began to rise, reaching 40.3°C and 40.4°C, and then began to decrease to a normal level. After the challenge, the body temperature of the adenovirus control group and the PBS control group was slightly higher than that of the other vaccine-immunized groups. The other experimental groups did not experience any increase in body temperature. In the early experiments of NADC34 strain infecting piglets, the body temperature of the blank control group could reach 41°C. The analysis is that the isolated strain is less pathogenic to adult pigs, so it did not cause very strong clinical reactions and histopathological damage. Necropsy of piglets and analysis of viral load on main tissues and organs: The experimental group had a lower viral load in tissues and organs than the adenovirus control group and the PBS control group. It shows that the recombinant adenovirus vaccine group has a better protection effect against the virus. Provide a theoretical basis for the prevention and control of the emergence of new PRRSV subtypes in China.

This study successfully constructed recombinant adenovirus pac-Ad5-34-GP3, pac-Ad5-34-GP5 and pac-Ad5-34-GP35 candidate vaccines and conducted immunogenicity tests against pigs. The recombinant adenovirus vaccine can effectively promote pig body fluids immunity and cellular immunity. The results of the challenge protection test showed that the vaccine immunization group reduced 2.5 titer compared with the control group. It shows that the recombinant adenovirus vaccine group has a better protection effect against the virus and provides vaccine reserves and theoretical support for the emergence of new PRRSV subtypes in China.

## Data Availability

The original contributions presented in the study are included in the article/supplementary material, further inquiries can be directed to the corresponding author/s.
